# Protective effects of *Sonchus asper* (L.) Hill, (Asteraceae) against CCl_4_-induced oxidative stress in the thyroid tissue of rats

**DOI:** 10.1186/1472-6882-12-181

**Published:** 2012-10-09

**Authors:** Rahmat Ali Khan

**Affiliations:** 1 Department of Biotechnology, Faculty of Sciences, University of Science and Technology, Bannu, Pakistan

**Keywords:** *Sonchus asper*, Oxidative stress, Antioxidant enzymes, Carbon tetrachloride, T_3_, T_4_

## Abstract

**Background:**

*Sonchus asper (L.) Hill, (Asteraceae)* is used in Pakistan as a traditional (“folk”) medicine for the treatment of hormonal disorders and oxidative stress. The present study was aimed to evaluate the efficacy of *Sonchus asper (L.) Hill, (Asteraceae)* methanolic extract (SAME) on hormonal dysfunction in thyroid tissue after carbon tetrachloride (CCl_4_)-induced oxidative stress.

**Methods:**

To examine the effects of SAME against the oxidative stress of CCl_4_ in thyroid tissue, 30 male albino rats were used. Protective effects of SAME were observed on thyroid hormonal levels, activities of antioxidant enzymes, lipid peroxidation (TBARS) and DNA damage.

**Results:**

Treatment with CCl_4_ significantly *(P<0.01)* reduced the levels of T_3_ and T_4_ and increased TSH levels. CCl_4_ exposure in rats reduced the activities of antioxidant enzymes but increased lipid peroxidation and DNA damage. Co-administration of SAME significantly (*P<0.01*) improved these alterations with respect to hormonal levels, activities of antioxidant enzymes and lipid peroxidation close to those seen in control rats.

**Conclusion:**

These results suggest that SAME can protect thyroid tissue against oxidative damage, possibly through the antioxidant effects of its bioactive compounds.

## Background

The thyroid gland secretes two types of iodine-containing amine hormones derived from the amino acid tyrosine: L-thyroxine (T_4_) and 3,5,3′L-triiodothyronine (T_3_). T_3_ and T_4_ are essential for the normal growth, development and function of organs. They also regulate the metabolism of hepatocytes whereas the liver metabolizes thyroid hormones. Hence, the liver and thyroid hormones are closely connected, and dysfunction of one causes a disturbance in the other [1].

Levels of thyroid hormones in blood are regulated by a negative feedback mechanism involving the hypothalamus, pituitary gland and thyroid gland. The hypothalamus releases thyrotrophic-releasing hormone (TRH). TRH stimulates the pituitary gland to release thyroid-stimulating hormone (TSH). TSH promotes thyroid cells to produce thyroid hormones. If the level of thyroid hormones is lower than that of TSH and the TRH level is high, trying to increase the level of thyroid hormones causes the risk of thyroid tumors in rats [2]. Propylthiouracil (PTU) and phenobarbital (PB) produce anti-thyroid effects that reduce the level of the thyroid hormones (T_3_ and T_4_) and increase the level of TSH [3,4]. CCl_4_ is a synthetic toxic chemical that increases lipid peroxidation [5,6]. CCl_4_ is converted into the trichloromethyl radical (^·^CCl_3_) and peroxytrichloromethyl radical (^·^OOCCl_3_). These free radicals then bind with polyunsaturated fatty acids (PUFAs) to generate lipid peroxides. Lipid peroxides are highly reactive, alter enzyme activities, and eventually induce injury or necrosis [7]. CCl_4_ induces oxidative DNA damage as well as causing the formation of DNA adducts, genetic mutations, strand breakage and chromosomal alterations (e.g., deletions and translocations) [8-10].

Various corticosteroids, vaccines and antiviral drugs have been used as chemotherapeutic agents, but have adverse side effects. Therefore, herbal products and traditional (“folk”) medicines which are more effective are used as substitutes for chemical agents. *Sonchus asper* (L.) Hill, (Asteraceae) is used for the treatment of bronchitis [11], gastrointestinal infection and cardiac dysfunction [12], kidney diseases [13] and cancer [14,15]. The effectiveness of this herb could be due to the presence of sesquiterpene lactone glycosides, ascorbic acid and carotenoids.

## Methods

### Plant collection

Plants of *Sonchus asper* maturity were collected from Wah cantt District Rawalpindi (Pakistan) during the month of June, 2011. Plants were identified and a specimen was submitted vide 147 at Herbarium of Pakistan, Quaid-i-Azam University Islamabad, Pakistan. Arial parts of plant (leaves, stem, flowers and seeds) were shade dried at room temperature for two weeks, chopped, grinded mechanically of mesh size 1 mm as described by Antonio et al. [16].

### Preparation of plant extract

1.5 kg *Sonchus asper* leaves powder was extracted in separatory funnel with 2.0 litre of absolute methanol with refluxing for 5 h. The extract was cooled at room temperature, filtered and evaporated under reduced pressure in rotary evaporator to *Sonchus asper* methanolic extract (SAME). SAME was stored at 4°C for *in vivo* studies [17].

### Animals

Six week old, 30 male albino rats (190–200 g) were provided by National Institute of Health Islamabad and were kept in ordinary cages at room temperature of 25 ± 3°C with a 12 h dark/light cycle. They were allowed to standard laboratory feed and water. The study protocol was approved by Ethical committee of Quaid-i-azam University Islamabad for laboratory animal feed and care.

### Experimental design

To study the antioxidant effects of SAME, male albino rats were equally divided into 05 groups (6 rats). Group 1 received only raw water and free access to food materials. Group II received CCl_4_ 3 ml/kg intraperitoneally in olive oil (Monday and Thursday). Group III and IV were given orally 100; 200 mg/kg b.w. (in DMSO), *Sonchus asper* methanolic extracts (SAME) after 48 h of CCl_4_ treatment (Wednesday and Saturday) as above. Groups V received only SAME in DMSO at a dose of 200 mg/kg b.w. (Wednesday and Saturday) [13]. After 24 h of the last treatment, all the animals were weighted, sacrificed; collected their blood, weighted and perfuse thyroid gland in ice-cold saline solution. Half of thyroid portion were treated with liquid nitrogen for further enzymatic and DNA damage analysis while the other portion was processed for histology.

### Assessment of serum thyroid hormones

Serum analysis of Various thyroid hormones’ such as T4, T3 and TSH were estimated by commercial radio amino assay kits 10227-Czch Republic (IM1447-IM3286), 10227-Czch Republic (IM1699-IM3287) and 10227-Czch Republic (IM3712-IM3713) Kit purchased from IMMUNOTECH Company respectively.

### Assessment of antioxidant enzymes

70 mg of thyroid tissue were homogenized in 10 volume of 100 mmol KH_2_PO_4_ buffer containing 1 mmol EDTA (pH 7.4) and centrifuged at 12,000 × g for 30 min at 4°C. The supernatant was collected and used for the following experiments as described below. Protein concentration was determined using crystalline BSA as standard.

### Catalase assay (CAT)

CAT activities were determined by the method of Chance and Maehly [18] with some modifications. The reaction solution of CAT activities contained: 2.5 ml of 50 mmol phosphate buffer (pH 5.0), 0.4 ml of 5.9 mmol H_2_O_2_ and 0.1 ml enzyme extract. Changes in absorbance of the reaction solution at 240 nm were determined after one minute. One unit of CAT activity was defined as an absorbance change of 0.01 as units/min.

### Peroxidase assay (POD)

Activities of POD were determined by the method of Chance and Maehly [18] with some modifications. The POD reaction solution contained: 2.5 ml of 50 mM phosphate buffer (pH 5.0), 0.1 ml of 20 mmol guaiacol, 0.3 ml of 40 mmol H_2_O_2_ and 0.1 ml enzyme extract. Changes in absorbance of the reaction solution at 470 nm were determined after one minute. One unit of POD activity was defined as an absorbance change of 0.01 units/min.

### Superoxide dismutase assay (SOD)

SOD activity of thyroid was estimated by the method of Kakkar et al. [19]. Reaction mixture of this method contained: 0.1 ml of phenazine methosulphate (186 μmol), 1.2 ml of sodium pyrophosphate buffer (0.052 mmol; pH 7.0), 0.3 ml of supernatant after centrifugation (1500 × g for 10 min followed by 10000 × g for 15 min) of lung homogenate was added to the reaction mixture. Enzyme reaction was initiated by adding 0.2 ml of NADH (780 μmol) and stopped after 1 min by adding 1 ml of glacial acetic acid. Amount of chromogen formed was measured by recording color intensity at 560 nm. Results are expressed in units/mg protein.

### Glutathione-S-transferase assay (GST)

Glutathione-S-transferase activity was assayed by the method of Habig et al. [20]. The reaction mixture consisted of 1.475 ml phosphate buffer (0.1 mol, pH 6.5), 0.2 ml reduced glutathione (1 mmol), 0.025 ml 1-chloro-2,4-dinitrobenzene (1 mmol CDNB) and 0.3 ml of homogenate in a total volume of 2.0 ml. The changes in the absorbance were recorded at 340 nm and enzymes activity was calculated as nmol CDNB conjugate formed/min/mg protein using a molar extinction coefficient of 9.6 × 10^3^M^-1^cm^-1^.

### Glutathione reductase assay (GR)

Glutathione reductase activity was determined by method of Carlberg and Mannervik [21]. The reaction mixture consisted of 1.65 ml phosphate buffer: (0.1 mol; pH 7.6), 0.1 ml EDTA (0.5 mmol), 0.05 ml oxidized glutathione (1 mmol), 0.1 ml NADPH (0.1 mmol) and 0.1 ml of homogenate in a total volume of 2 ml. Enzyme activity was quantitated at 25°C by measuring disappearance of NADPH at 340 nm and was calculated as nmol NADPH oxidized/min/mg protein using molar extinction coefficient of 6.22 × 10^3^ M^-1^cm^-1^.

### Glutathione peroxidase assay (GSH-Px)

Glutathione peroxidase activity was assayed by the method of Mohandas et al. [22]. The reaction mixture consisted of 1.49 ml phosphate buffer (0.1 mol; pH 7.4), 0.1 ml EDTA (1 mmol), 0.1 ml sodium azide (1 mmol), 0.05 ml glutathione reductase (1 IU/ml), 0.05 ml GSH (1 mmol), 0.1 ml NADPH (0.2 mmol), 0.01 ml H_2_O_2_ (0.25 mmol) and 0.1 ml of homogenate in a total volume of 2 ml. The disappearance of NADPH at 340 nm was recorded at 25°C. Enzyme activity was calculated as nmol NADPH oxidized/min/mg protein using molar extinction coefficient of 6.22 × 10^3^ M^-1^cm^-1^.

### Î³-glutamyl transpeptidase assay (Î³-GT)

This was determined by the method of Orlowski and Meister [23] using glutamyl *p*-nitroanilide as substrate. The reaction mixture in a total volume of 1.0 ml contained 0.2 ml of homogenate which was incubated with 0.8 ml substrate mixture (containing 4 mmol glutamyl *p*-nitroanilide, 40 mmol glycylglycine and 11 mmol MgCl_2_ in 185 mmol Tris–HCl buffer, pH 8.25) at 37°C. Ten minutes after initiation of the reaction, 1.0 ml of 25% TCA was added and mixed to terminate the reaction. The solution was centrifuged and the supernatant fraction was read at 405 nm. Enzyme activity was calculated as nmol *p*-nitroaniline formed/min/mg protein using a molar extinction coefficient of 1.74 × 10^3^ M^-1^cm^-1^.

### Quinone reductase assay

The activity of quinone reductase was determined by the method of Benson et al. [24]. The 3.0 ml reaction mixture consisted of 2.13 ml Tris–HCl buffer (25 mmol; pH 7.4), 0.7 ml BSA, 0.1 ml FAD, 0.02 ml NADPH (0.1 mmol), and 0.l ml of homogenate. The reduction of dichlorophenolindophenol (DCPIP) was recorded at 600 nm and enzyme activity was calculated as nmol of DCPIP reduced/min/mg protein using molar extinction coefficient of 2.1 × 10^4^ M^-1^cm^-1^.

### Reduced glutathione assay (GSH)

Reduced glutathione was estimated by the method of Jollow et al. [25]. 1.0 ml sample of homogenate was precipitated with 1.0 ml of (4%) sulfosalicylic acid. The samples were kept at 4°C for 1 h and then centrifuged at 1200 × g for 20 min at 4°C. The total volume of 3.0 ml assay mixture contained 0.1 ml filtered aliquot, 2.7 ml phosphate buffer (0.1 mol; pH 7.4) and 0.2 ml DTNB (100 mmol). The yellow color developed was read immediately at 412 nm on a SmartSpecTM plus Spectrophotometer. It was expressed as μmol GSH/g tissue.

### Estimation of lipid peroxidation assay (TBARS)

The assay for lipid peroxidation was carried out following the method of Wright et al. [26]. The reaction mixture in a total volume of 1.0 ml contained 0.58 ml phosphate buffer (0.1 mol; pH 7.4), 0.2 ml homogenate sample, 0.2 ml ascorbic acid (100 mmol), and 0.02 ml ferric chloride (100 mmol). The reaction mixture was incubated at 37°C in a shaking water bath for 1 h. The reaction was stopped by addition of 1.0 ml 10% trichloroacetic acid. Following addition of 1.0 ml 0.67% thiobarbituric acid, all the tubes were placed in boiling water bath for 20 min and then shifted to crushed ice-bath before centrifuging at 2500 × g for 10 min. The amount of TBARS formed in each of the samples was assessed by measuring optical density of the supernatant at 535 nm using spectrophotometer against a reagent blank. The results were expressed as nmol TBARS/min/mg tissue at 37°C using molar extinction coefficient of 1.56 ×10^5^ M^-1^cm^-1^.

### Hydrogen peroxide assay (H_2_O_2_)

Hydrogen peroxide (H_2_O_2_) was assayed by H_2_O_2_-mediated horseradish peroxidase-dependent oxidation of phenol red by the method of Pick and Keisari [27]. 2.0 ml of homogenate sample was suspended in 1.0 ml of solution containing phenol red (0.28 nmol), horse radish peroxidase (8.5 units), dextrose (5.5 nmol) and phosphate buffer (0.05 mol; pH 7.0) and were incubated at 37°C for 60 min. The reaction was stopped by the addition of 0.01 ml of NaOH (10 N) and then centrifuged at 800 × g for 5 min. The absorbance of the supernatant was recorded at 610 nm against a reagent blank. The quantity of H_2_O_2_ produced was expressed as nmol H_2_O_2_/min/mg tissue based on the standard curve of H_2_O_2_ oxidized phenol red.

### Nitrite assay

Nitrite assay was conducted by using Griess reagent. Tissue and serum samples were deproteinized by equal volumes of 0.3 mol NaOH and 5% ZnSO_4_ and centrifuged at 6400 × g for 20 min and supernatant was collected. 1.0 ml of Griess reagent was added into the cuvette and blanks the spectrophotometer at 540 nm. Then 20 μl supernatant was added in cuvette containing Griess Reagent. Nitrite concentration was calculated using a standard curve for sodium nitrite.

### DNA fragmentation % assay

DNA fragmentation % assay was conducted using the procedure of Wu et al. [28] with some modifications. The thyroid tissue (50 mg) was homogenized in 10 volumes of a TE solution pH 8.0 (5 mmol Tris–HCl, 20 mmol EDTA) and 0.2% triton X-100. 1.0 ml aliquot of each sample was centrifuged at 27,000 × g for 20 min to separate the intact chromatin (pellet, B) from the fragmented DNA (supernatant, T). The pellet and supernatant fractions were assayed for DNA content using a freshly prepared DPA (Diphenylamine) solution for reaction. Optical density was read at 620nm at (SmartSpecTM Plus Spectrophotometer catalog # 170–2525) spectrophotometer. The results were expressed as amount of % fragmented DNA by the following formula;

(1) %FragmentedÂ DNA=T×100/T+B

### Histopathalogical determination

For microscopic evaluation thyroid tissues were fixed in a fixative (Absolute alcohol 60%, Formaldehyde 30%, Glacial acetic acid 10%) and embedded in paraffin, sectioned at 4 μm and subsequently stained with hematoxylin/eosin. Sections were studied under light microscope (DIALUX 20 EB) at 40 magnifications. Slides of all the treated groups were studied and photographed. A minimum 12 fields of each section were studied and approved by pathologist without saying of its treatment nature.

### Statistical analysis

To determine the treatment effects one way analysis of variance was carried by computer software SPSS 13.0. Level of significance among the various treatments was determined by LSD at 0.05% level of probability.

## Results

### Effect of SAME on thyroid hormones TSH, T3, T4 level

The effects of SAME on hormonal level of thyroid glands viz TSH, T_3_ and T_4_ are shown in Table 1. CCl_4_ administration significantly *(P<0.01)* reduced the level of T_3_ and T_4_ and increased TSH. Administration of SAME significantly *(P<0.01)* recovered the level of thyroid hormones dose dependently.

**Table 1:** Effect of SAME on thyroid hormones TSH, T3, T4 levels

**Treatment**	**TSH**	**T4**	**T3**
	**ng/dl**	**ng/ml**	**ng/ml**
Control	15.50±1.23++	6.233±0.357++	59.83±2.24++
3 ml/kg CCl_4_	26.17±1.14**	3.800±0.221**	44.17±1.40**
100 mg/kg SAME+CCl_4_	20.17±1.1**++	4.983±0.289**++	49.17±1.14**+
200 mg/kg SAME+CCl_4_	16.00±1.03++	5.933±0.278++	57.83±1.82++
200 mg/kg SAME alone	15.17±1.22++	6.217±0.244++	58.33±1.73++

Mean ±SE (n=6 number).

** indicate significance from the control group at *P<0.01* probability level.

++ indicate significance from the CCl_4_ group at *P<0.01* probability level.

### Effect of SAME on tissue protein and activities of the antioxidant enzymes CAT, POD and SOD

Changes in the levels of tissue protein and the antioxidant enzymes CAT, POD and SOD in all the experimental groups of rats are shown in Table 2. Administration of CCl_4_ significantly *(P<0.01)* decreased the amount of protein as well as the activities of the antioxidant enzymes CAT, POD and SOD as compared with the control group. Co-administration of SAME significantly *(P<0.01)* recovered the toxicity of CCl_4_ in thyroid tissue in a dose-dependent manner.

**Table 2:** Effect of SAME on thyroid hormones tissue protein, CAT, POD, SOD activities

**Treatment**	**Protein (ug/mg tissue)**	**CAT**	**POD**	**SOD**
		**(U/min)**	**(U/min)**	**(U/mg protein)**
Control	0.65±0.010++	6.92±0.23++	4.92±0.015++	12.58±0.218++
3 ml/kg CCl_4_	0.33±0.010**	4.02±0.062**	2.81±0.0548**	6.78±0.248**
100 mg/kg SAME+CCl_4_	0.49±0.010**++	5.35±0.36**++	3.79±0.0601**++	10.11±0.349**++
200 mg/kg SAME+CCl_4_	0.63±0.009++	6.36±0.15++	4.76±0.061++	11.75±0.470++
200 mg/kg SAME alone	0.66±0.016++	6.95±0.071++	4.92±0.065++	13.00±0.211++

Mean ±SE (n=6 number).

** indicate significance from the control group at *P<0.01* probability level.

++ indicate significance from the CCl_4_ group at *P<0.01* probability level.

### Effect of SAME on thyroid levels of GST, GR, GSH-Px, GGT and QR

The protective effect of SAME on the activities of the enzymes GST, GR, GSH-Px, γ-GT, and quinone reductase is shown in Table 3. Compared with the control group, CCl_4_ treatment in rats significantly *(P<0.01)* decreased the activity of GST, GSR, GSH-Px and quinone reductase, whereas the activity of γ-GT was increased.

**Table 3:** Effect of SAME on thyroid GST, GSR, GSHpx, GGT, QR

**Treatment**	**GST nM**	**GSR nM**	**GSHpx nM**	**γ-GT**	**QR nM**
	**/min/mg protein**	**/min/mg protein**	**/min/mg protein**	**(nM/min/mg protein)**	**/min/mg protein**
Control	95.67±2.03++	59.84±2.30 ++	41.67±0.97++	2.99±0.45++	149.11±2.00++
3 ml/kg CCl_4_	46.00±0.8**	34.96±1.44**	18.74±0.49**	5.98±0.14**	87.50±1.48**
100 mg/kg SAME+CCl_4_	60.83±1.42**++	50.84±1.17**++	29.34±0.85**++	3.98±0.25**++	110.41±1.73**++
200 mg/kg SAME+CCl_4_	87.67±2.70++	56.51±1.74++	38.34±1.42 ++	3.05±0.16++	146.63±1.16 ++
200 mg/kg SAME alone	95.17±2.83++	61.51±1.97++	42.34±1.38 ++	3.00±0.07++	150.46±1.42++

Mean ±SE (n=6 number).

** indicate significance from the control group *P<0.01* probability level.

++ indicate significance from the CCl_4_ group at *P<0.01* probability level.

Co-treatment of CCl_4_-intoxicated rats with SAME markedly decreased the levels of γ-GT but increased the activity of GST, GR, GSH-Px and quinone reductase in a dose-dependent manner.

### Effect of SAME on thyroid levels of GSH, TBARS, H_2_O_2_, and nitrites

Changes in the tissue content of TBARS, H_2_O_2_, GSH and nitrites are shown in Table 4. Compared with the control group, administration of CCl_4_ significantly decreased *(P<0.01)* the levels of GSH but significantly increased the levels of nitrites, H_2_O_2_, and TBARS contents. Co-administration of SAME significantly *(P<0.01)* increased the levels of GSH and decreased TBARS levels at both doses, whereas H_2_O_2_ and nitrite contents were ameliorated significantly *(P<0.01)* only at 200 mg/kg of SAME. However, administration of SAME alone did not show significant changes as compared with the control group.

**Table 4:** **Effect of SAME on thyroid GSH, TBARS, H**_**2**_**O**_**2**_**, nitrite**

**Treatment**	**GSH (μM/g tissue)**	**TBARS(nM /min/mg protein)**	**H**_**2**_**O**_**2**_**(nM/min/mg tissue)**	**Nitrite (μM/ml)**
Control	4.590±0.29++	9.66±.66++	6.5±0.48++	48.0±2.38++
3 ml/kg CCl_4_	2.82±0.06**	19.02±0.44**	11.3±1.05**	62.0±2.14**
100 mg/kg SAME+CCl_4_	4.06±0.19**++	14.33±0.49**++	10.0±0.36**	52.8±2.85**++
200 mg/kg SAME+CCl_4_	4.50±0.20++	13.16±0.60**++	6.5±0.48++	44.2±2.06++
200 mg/kg SAME alone	4.73±0.198++	11.33±0.76++	6.13±0.38++	41.3±2.09 ++

Mean ±SE (n=6 number).

** indicate significance from the control group at *P<0.01* probability level.

++ indicate significance from the CCl_4_ group at *P<0.01* probability level.

### Effect of SAME on thyroid % DNA, tissue weight (TW), and relative tissue weight (RTW)

Protective effects of SAME against CCl_4_ administration on rat thyroid weight, relative tissue weight and % DNA fragmentation by DPA methods are shown in Table 5. Administration of CCl_4_ significantly increased *(P<0.01)* thyroid weight and relative thyroid weight and % DNA when compared to control group. DNA damages was significantly *(P<0.01)* recovered by SAME. Tissue weight and relative tissue weight of thyroid gland was significantly *(P<0.01)* decreased to normal levels at dose dependent manner. Non significant variation was found with SAME alone*.*

**Table 5:** Effect of SAME on thyroid % DNA, TW, RTW

**Treatment**	**DNA %**	**TW mg**	**RTW**
Control	48.83±2.32 ++	55.83±2.70++	0.553±0.02++
3 ml/kg CCl_4_	68.64±2.05**	79.67±2.29**	0.797±0.09**
100 mg/kg SAME+CCl_4_	57.25±1.80 **++	67.83±2.44**++	0.673±0.03**++
200 mg/kg SAME+CCl_4_	49.40±3.80++	57.83±2.06++	0.573±0.06++
200 mg/kg SAME alone	48.33±2.62++	56.17±2.52++	0.567±0.05++

Mean ±SE (n=6 number).

** indicate significance from the control group at *P<0.01* probability level, respectively.

++ indicate significance from the CCl_4_ group at *P<0.01* probability level, respectively.

### Effect of SAME on the histopathology of thyroid tissue

**Figure 1:**
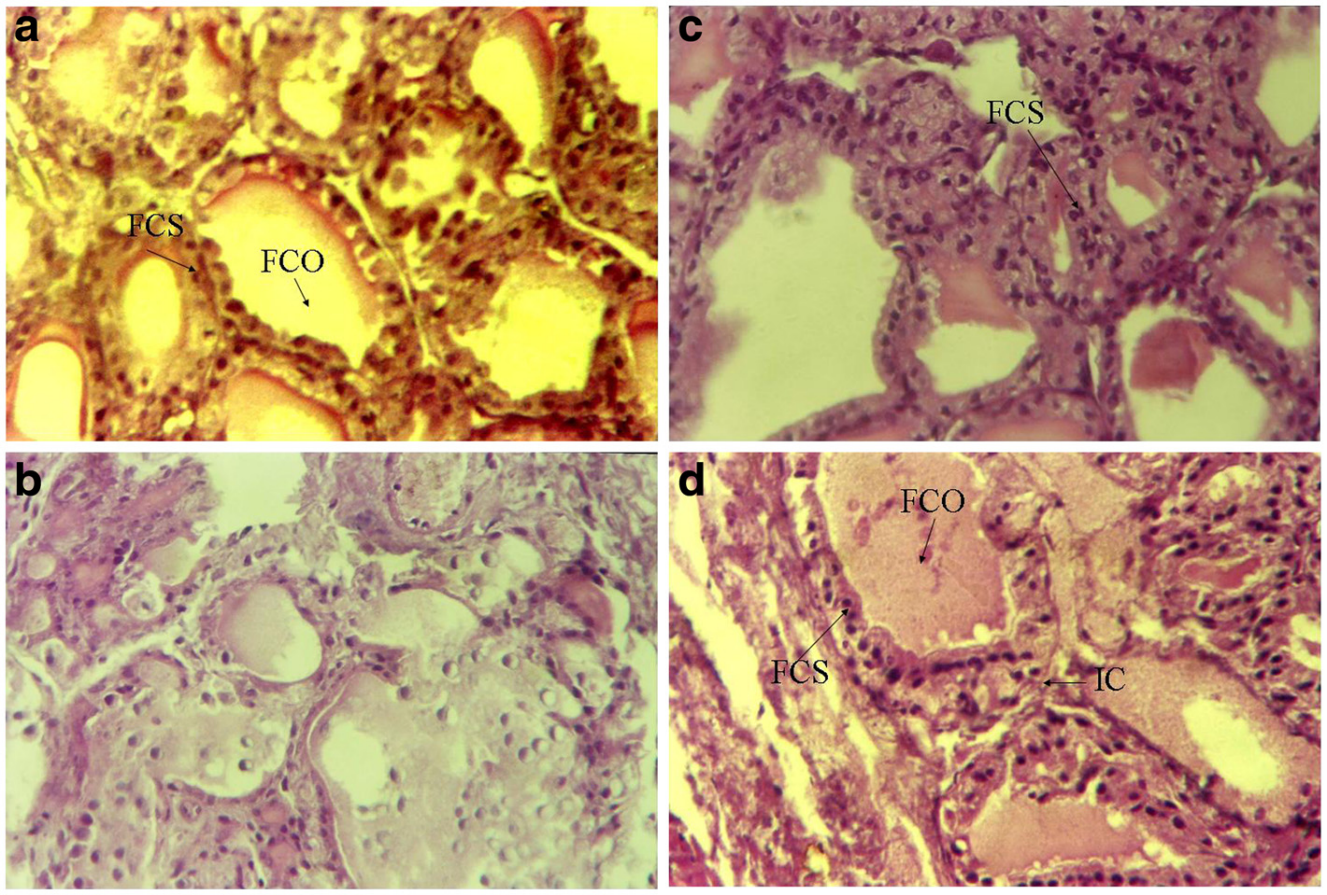
**(1a) Hemotoxylin and Eosin-stained thyroid section of control rat (1b) CCl**_**4**_**(1c) CCl**_**4**_**+ 100 mg/kg SAME (1d) CCl**_**4**_**+ 200 mg/kg SAME.**

Administration of CCl_4_ caused significant colloid depletion and hypertrophy. Congestion in blood vessels and degradation of follicular shape caused hyperplasia in follicular cells (Figure 1). Co-treatment with 100 mg/kg and 200 mg/kg SAME significantly reversed the injuries close to those seen in control rats. Rats administered SAME showed normal histology with mild congestion in blood vessels (Table 6).

**Table 6:** Effect SAME on thyroid histopathology

**Treatment**	**Colloids depletion**	**Hypertrophy**	**Hyperplasia**	**Blood vessel congestion**	**Follicular architecture**
Control	-	-	-	-	-
3 ml/kg CCl_4_	+++	+++	+++	+++	+++
100 mg/kg SAME+CCl_4_	-	−/+	-	−/+	−/+
200 mg/kg SAME+CCl_4_	-	-	-	-+	-
200mg/kg SAME alone	-	-	-	-	-

-, normal; −/+, mild; ++, medium; +++, severely damaged.

## Discussion

The present study was conducted to ascertain the *in-vivo* protective effect of SAME against CCl_4_-induced thyroid toxicity in rats. Our results showed that administration of CCl_4_ depleted the levels of thyroid hormones (i.e., secretion of T_3_ and T_4_) and elevated the TSH level in the serum of rats, effects that were reversed by SAME administration. Similar results have been reported by the administration of 10 mg/kg PTU and 100 mg/kg PB in rats, which caused depletion of thyroid hormones and elevated the levels of TSH [4]. Antioxidant enzymes have key roles in detoxification of the free radicals and reactive oxygen species (ROS) produced during exposure to toxic chemicals and metabolism of various xenobiotics. Our results showed that CCl_4_ intoxication caused the depletion of tissue protein and activities of the antioxidant enzymes CAT, POD and SOD. Co-administration of SAME markedly erased the toxicity of CCl_4_ and the enzymatic activities of CAT, POD and SOD towards the normal range. The ameliorating effects of SAME against the toxicity of CCl_4_ might be due to the presence of ascorbic acid, sesquiterpenoids, flavonoids and saponins [29]. The glutathione system includes glutathione, glutathione reductase, glutathione peroxidases and glutathione S-transferases. We showed here that treatment with CCl_4_ decreased the activities of GST, GR, GSH-Px and quinone reductase, whereas the activity of γ-GT was increased. Decreases in the activity of GST might be due to the decreased availability of GSH content and increased lipid peroxidation. Co-administration of SAME erased the intoxication caused by. Similar observations were reported by Sreelatha et al. [30]. GSH is an important protein thiol that plays the main part in the catalysis of various metabolic activities and coordinates the defense system of the body against oxidative stress. Deficiency of GSH within living cells causes various disorders, oxidative stress and cellular injuries. Administration of CCl_4_ decreased GSH contents but increased the levels of nitrites, H_2_O_2_, and TBARS as compared with the control group. Nitrites are produced in livers treated with CCl_4,_ which in turn are converted into peroxynitrites in the acidic medium of CCl_4._ These peroxynitrite anions oxidize biomolecules, which eventually leads to lipid peroxidation [31]. Co-administration of rutin and SAME significantly recovered GSH-decreased TBARS, H_2_O_2_ and nitrite contents. Similar observations were observed by Srinivasan et al. [32] upon chronic administration of CCl_4_ in the livers and kidneys of rats. The present study showed that CCl_4_ increased tissue weight, relative tissue weight, and % DNA fragmentation, which was also revealed by the DNA ladder assay [9]. Similar observations were observed by Rodriguez et al. [33] in rats with 2-month treatments with 1% potassium perchlorate. Administration of CCl_4_ caused significant colloid depletion and hypertrophy, blood-vessel congestion, and hyperplasia of follicular cells. Co-treatment with SAME significantly erased such injuries close to those seen in control rats. Similar observations were reported by Hooth et al. [34] during exposure to drinking water containing sodium chlorate in rats.

## Conclusion

These results demonstrate that administration of SAME may be useful in the treatment and prevention of thyroid oxidative stress associated abnormalities.

## Abbreviations

GSH: Reduced glutathione; GSSG: Oxidized glutathione; BSA: Glutathione reductase gamma-glutamyl p-nitroanilide, glycylglycine, bovine serum albumin; DTNB: 1,2-dithio-bis nitro benzoic acid; CDNB: 1-chloro-2,4-dinitrobenzene; NADPH: Reduced nicotinamide adenine dinucleotide phosphate; FAD: CCl_4_ flavine adenine dinucleotide; TBA: glucose-6-phosphate Tween-20, 2,6-dichlorophenolindophenol, thiobarbituric acid; TCA: Picric acid sodium tungstate, sodium hydroxide, trichloroacetic acid; PCA: Perchloric acid.

## Competing interests

The author declares that they have no competing interests.

## Author’s contributions

RAK made a significant contribution to design, analyses of data and drafting of the manuscript. The author read and approved the final manuscript.

## Pre-publication history

The pre-publication history for this paper can be accessed here:

http://www.biomedcentral.com/1472-6882/12/181/prepub
